# Acoustic Assessment of Tone Production of Prelingually-Deafened Mandarin-Speaking Children With Cochlear Implants

**DOI:** 10.3389/fnins.2020.592954

**Published:** 2020-11-04

**Authors:** Yitao Mao, Hongsheng Chen, Shumin Xie, Li Xu

**Affiliations:** ^1^Department of Radiology, Xiangya Hospital, Central South University, Changsha, China; ^2^Department of Otolaryngology-Head and Neck Surgery, Xiangya Hospital, Central South University, Changsha, China; ^3^Communication Sciences and Disorders, Ohio University, Athens, OH, United States

**Keywords:** cochlear implant, tone production, Mandarin Chinese, lexical tone, acoustic analysis, pediatric

## Abstract

**Objective:**

The purpose of the present study was to investigate Mandarin tone production performance of prelingually deafened children with cochlear implants (CIs) using modified acoustic analyses and to evaluate the relationship between demographic factors of those CI children and their tone production ability.

**Methods:**

Two hundred seventy-eight prelingually deafened children with CIs and 173 age-matched normal-hearing (NH) children participated in the study. Thirty-six monosyllabic Mandarin Chinese words were recorded from each subject. The fundamental frequencies (F0) were extracted from the tone tokens. Two acoustic measures (i.e., differentiability and hit rate) were computed based on the F0 onset and offset values (i.e., the tone ellipses of the two-dimensional [2D] method) or the F0 onset, midpoint, and offset values (i.e., the tone ellipsoids of the 3D method). The correlations between the acoustic measures as well as between the methods were performed. The relationship between demographic factors and acoustic measures were also explored.

**Results:**

The children with CIs showed significantly poorer performance in tone differentiability and hit rate than the NH children. For both CI and NH groups, performance on the two acoustic measures was highly correlated with each other (*r* values: 0.895–0.961). The performance between the two methods (i.e., 2D and 3D methods) was also highly correlated (*r* values: 0.774–0.914). Age at implantation and duration of CI use showed a weak correlation with the scores of acoustic measures under both methods. These two factors jointly accounted for 15.4–18.9% of the total variance of tone production performance.

**Conclusion:**

There were significant deficits in tone production ability in most prelingually deafened children with CIs, even after prolonged use of the devices. The strong correlation between the two methods suggested that the simpler, 2D method seemed to be efficient in acoustic assessment for lexical tones in hearing-impaired children. Age at implantation and especially the duration of CI use were significant, although weak, predictors for tone development in pediatric CI users. Although a large part of tone production ability could not be attributed to these two factors, the results still encourage early implantation and continual CI use for better lexical tone development in Mandarin-speaking pediatric CI users.

## Introduction

The modern cochlear implant (CI) is currently the most successful neural prosthesis in wide clinical application. It can restore the sense of hearing for hearing-impaired individuals by bypassing the damaged sensory cells in the inner ear and stimulating the auditory nerve directly (Wilson, 2019). Previous evidence showed that severely to profoundly deafened children obtained enormous benefits for their speech and language development after cochlear implantation (Niparko et al., 2010). Speech production ability and language development of CI users, especially of those children with prelingual deafness, have been the major focus of the postoperative rehabilitation process. Detailed acoustic analyses of the production of vowels and fricatives in CI children have shown significant progress in phoneme development, but there are still significant remaining deficits in speech production in those children (Uchanski and Geers, 2003; Yang et al., 2015, 2017a; Yang and Xu, 2017). For CI children who speak tonal languages, such as Mandarin Chinese, speech production is compounded by the involvement of lexical tones. In tonal languages, the word meaning depends not only on the phonemes (such as the combination of consonants and vowels), but also on the pattern of tones (i.e., the fundamental frequency, F0) of the syllables. In other words, for a specific syllable, changing the F0 contour will bring about a change in the meaning of the syllable. There are four tones in Mandarin, namely tones 1, 2, 3, and 4. The F0 contours are (1) high and flat, (2) low at the beginning and then rising, (3) falling at the beginning and then rising with a dip in the middle, and (4) high-falling, respectively. Such tonal information, primarily carried by the F0, is not adequately coded in current CI devices (Han et al., 2009; Xu and Zhou, 2011; Limb and Roy, 2014; Deroche et al., 2019). Previous studies that focused on tone perception have revealed significant deficits of CI children in tone recognition tasks, with tremendous variability observed across CI subjects (Lee et al., 2002; Peng et al., 2004; Han et al., 2009; Zhou et al., 2013; Mao and Xu, 2017; Holt et al., 2018). As a result of tone recognition deficits, the tone production ability of prelingually deafened children with CIs might also be compromised.

The specific mechanism of the influence of auditory feedback on oral speech is not entirely clear. It was suggested that auditory feedback has a significant and immediate effect on oral speech (Davidson, 1959; Lane and Tranel, 1971). For example, when exposed to noise, the talker’s vocal intensity would increase involuntarily, a phenomenon known as the “Lombard effect” (Lane and Tranel, 1971). If the auditory feedback is deliberately delayed, it will cause the speech speed to slow down (Davidson, 1959). The frequency information is also affected by the auditory feedback frequency (Elman, 1981). For phoneme pronunciation, Houde and Jordan (1998) found that if the first three formant frequencies of vowels in auditory feedback are deliberately changed, the produced vowels would be unconsciously replaced by other vowels to compensate for these formant changes. All these findings supported the hypothesis that there is a closely coupled loop between auditory perception and vocal production (Deroche et al., 2019), and auditory feedback can regulate oral speech instantaneously (Natke and Kalveram, 2001; Amir et al., 2003; Mora et al., 2012; Liu et al., 2020).

For postlingually deafened adults, the connection between perception and production will decline gradually due to the loss of auditory feedback, whereas for prelingually deafened children, this connection has not been established. Earlier studies have shown that for postlingually deafened adults, the loss of hearing does not have a significant impact on their speech intelligibility but only gradually changes some acoustic parameters of their oral speech with a very slow rate (Waldstein, 1990; Leder and Spitzer, 1993; Plant, 1993). In the absence of auditory feedback, they seem to use their knowledge and experiences to regulate their vocal organs to make the desired sound (Matthies et al., 1994). However, for children with prelingual deafness, the connection between hearing and vocal production has not been well established in their speech acquisition stage, which harms their speech intelligibility. Because of the absence of effective auditory feedback, prelingually deafened children would likely rely on visual or somatosensory inputs to establish a feedback connection with their vocal production (Tobey et al., 1991; Osberger et al., 1993; Nava et al., 2014; Selleck and Sataloff, 2014). With their auditory function partially restored with CIs, prelingually deafened children still face challenges in their speech production (Uchanski and Geers, 2003; Yang et al., 2015, 2017a; Yang and Xu, 2017).

Pitch information is not adequately coded in the contemporary envelope-based speech processing strategies in which fixed-rate electrical stimulations delivered to a small number of CI electrodes result in poor pitch perception in CI users (Wilson and Dorman, 2008; Xu and Zhou, 2011). At present, numerous studies have reported that there are considerable deficits in Mandarin tone recognition for CI children (see Tan et al., 2016; Chen and Wong, 2017; Liu et al., 2017 for reviews). For example, Zhou et al. (2013) and Mao and Xu (2017) reported that CI children achieved Mandarin tone recognition of 67.3 to 82.3% correct, whereas their normal-hearing (NH) counterpart obtained > 95% correct. The inadequate tonal information since childhood is likely to make tone production problematic in the speech development of those CI children who use Mandarin Chinese as their mother tongue. Several previous studies with relatively small sample sizes have found that the tone production ability of Mandarin-speaking pediatric CI users was significantly poorer compared with NH children at a similar age range (Wei et al., 2000; Peng et al., 2004; Xu et al., 2004, 2011; Han et al., 2007; Zhou et al., 2013; Tang et al., 2019). Peng et al. (2004) reported tone production accuracy in 30 CI children aged between 6.0 and 12.5 years old based on the subjective judgment of NH adults. The average tone production accuracy was only 53.1% correct. Zhou et al. (2013) also reported that tone intelligibility was only 46.8% correct for their 76 CI children with an age range of 2.4–16.2 years old. A common finding by these studies was that these CI children had tremendous individual variability in tone production ability and that their tone production was distributed from the chance level to near-perfect performance.

With the increasing number of prelingually deaf children who have received cochlear implantation in China in the past decades, it is of great importance to explore their vocal tone ability using a more objective way of evaluation. In our previous studies (Xu et al., 2007; Zhou et al., 2013; Mao et al., 2017), we used an artificial neural network to evaluate the tone production ability of children with CIs. The artificial neural network yielded an objective and efficient way to assess tone production ability; however, it could not reveal what the deficits in tone production were in CI children. Acoustic analyses might be of great value in pinpointing such deficits. Barry and Blamey (2004) proposed a method of acoustic analysis to assess Cantonese tone production. Zhou and Xu (2008) modified this method and applied it in Mandarin tone production evaluation of CI children. This acoustic method was based on the F0 contours of the produced tone tokens. In particular, the onset and offset frequencies of the F0 contours were extracted, and the tonal ellipses were generated over the scatter plots of the F0 onset versus F0 offset values. The spread and degree of overlap among tonal ellipses were quantified by a series of acoustic indices to reflect various aspects of the tone production ability. In a recent study, Tang et al. (2019) examined the F0 contours of tone tokens produced by prelingually deafened children with CIs. The authors quantified tone production accuracy based on the curvature of the F0 contours. In the 72 pediatric CI users, those who received CIs between 1 and 2 years of age demonstrated near-normal tone contours, whereas all other CI children’s tone patterns tended to be flattened (Tang et al., 2019).

In the present study, we recruited a large cohort of prelingually deafened children with CIs (*N* = 278) and age-matched NH children (*N* = 173). A modified acoustic analysis method was developed and used to evaluate the tone production skills of the children. The purpose is to verify the effectiveness of this modified acoustic analysis method in the evaluation of tone production of pediatric CI users and to explore the different tone-production characteristics of the hearing-impaired group from those of the NH group. Correlational analyses were implemented between several demographic factors of the CI group and the acoustic indices obtained by our modified method in the present study. In addition, a generalized linear model (GLM; Song et al., 2013) was also used to explore further the effects of demographic factors on tone production performance.

## Materials and Methods

### Subjects

A total of 278 prelingually deafened, Mandarin-speaking children were recruited to participate in the present study. The inclusion criteria were as follows: (1) prelingual sensorineural hearing loss, (2) bilateral severe to profound hearing loss (≥85 dB HL) and implanted unilaterally, (3) limited or no hearing aid use experiences before CI implantation, (4) chronological age was >2 years old, (5) the age at implantation was <12 years old, (6) using Mandarin as the mother tongue or the rehabilitation language, and (7) hearing impairment was the only health problem. In this CI group, there were 152 boys and 126 girls, ranging in chronological age from 2.13 to 19.04 (mean ± SD: 6.64 ± 3.46) years old, the age at implantation was from 0.50 to 11.02 (3.38 ± 2.25) years old, and the CI use duration was from 0.14 to 11.20 (3.26 ± 2.64) years.

As the control group, 173 Mandarin-speaking NH children from kindergartens and primary schools with ages between 2.28 and 12.51 (6.83 ± 2.85) years old were recruited in the present study. The parents reported the NH status. In the NH group, there were 94 boys and 79 girls. The mean chronological ages of these two groups were not statistically different (*t*-test, *t* = 0.479, *p* > 0.05). The use of human subjects was reviewed and approved by the Institutional Review Board of Ohio University.

### Test Materials

Eighteen monosyllables (i.e., bei, bi, chi, chuang, deng, hu, jian, mao, mi, qiang, san, shu, tang, tu, wa, wu, ye, and yu) in Mandarin Chinese were selected as the targets. Each monosyllable was assigned two tones to make up a tone contrast. Therefore, the test materials consisted of 36 Chinese words (a complete list of the 36 words can be found in Han et al., 2009). All the 36 words were at the vocabulary level of young children and were used in previous studies (Han et al., 2009; Zhou et al., 2013). Each of the tone contrasts (i.e., tone 1 vs. 2, tone 1 vs. 3, tone 1 vs. 4, tone 2 vs. 3, tone 2 vs. 4, and tone 3 vs. 4) had three pairs of monosyllabic words, and each tone type (i.e., tone 1, tone 2, tone 3, and tone 4) had nine monosyllabic words, thus making it balanced among the number of monosyllabic words for tone contrasts or tone types.

### Test Procedure

The test was conducted in a sound-treated room. The 36 test words were presented to the subjects in the form of cards used to elicit vocal production. Each card displayed a picture illustrating the meaning of the target word, the Chinese character, and the corresponding Pinyin (i.e., an alphabetic form indicating the pronunciation of the Chinese character). The experimenter first explained the test requirements to the subjects to make sure they understood the tasks. A recorder microphone was then placed in front of the subjects with a distance of approximately 10 cm from the subject’s lips. With the help of test cards, the experimenter guided the subjects to speak out the target words, which were recorded at a sampling rate of 44.1 kHz and an amplitude resolution of 16 bits.

### Acoustic Analysis

An autocorrelation algorithm was used to extract the F0s of each produced tone token (Xu et al., 2006, 2007; Zhou et al., 2008). The F0 contours were then drawn on a narrowband spectrogram for accuracy comparison. Occasionally, there were some errors in the extracted F0 contours, which, for a large part, were doubling and halving errors. Those errors were corrected manually on the spectrogram.

To eliminate the impact of individual vocal pitch on the differentiability of the four tones when the data were pooled together across all subjects, the F0 data were normalized subject by subject. The normalization algorithm was as follows: (1) we took the mean F0 of all tokens in tone 1 of one subject and called it *M*, (2) all F0 data of this subject was converted to semitones based on the equation below, and (3) the normalization was then applied for all subjects in both groups.

$$\text{Semitone}=12\times {\text{log}}_{2}\,\left(\frac{\text{F}\,0}{M}\right)$$

In Zhou and Xu (2008), the F0 onset and offset of the F0 contours were extracted, and four tonal ellipses based on the four scatter plots of F0 onset versus F0 offset data of the four tones were defined. The center of the ellipses was the center of the scatter distribution, and the major and minor axes of the ellipses were of two standard deviations (SDs) of the distribution in length. Three acoustic indices were calculated based on the tonal ellipses. Index 1 was defined as the ratio of the area of quadrangle formed by joining the centers of the four tonal ellipses relative to the averaged area of the four ellipses. Index 2 was defined as the ratio of the averaged distance of the centers of the four tonal ellipses from each other relative to the averaged lengths of the two axes for four Mandarin tonal ellipses. Index 3 was the averaged proportion of the number of points of a specific tone inside that specific tonal ellipse. The three indices were found highly correlated with each other, with all correlation coefficients >0.94. In the present study, we modified these indices into two features: tone differentiability and tone hit rate. In addition, besides the two endpoints on the F0 contours, we also incorporated the middle point of the F0 contours in our computation to capture potentially distinctive characteristics of tone contour, which could be especially meaningful for tone 3 (Tupper et al., 2020). This latter method was referred to as the three-dimensional (3D) method to differentiate it from the 2D method that used the F0 onset and offset values only in the present study.

#### Tone Differentiability

We modified the algorithm of the Index 1 and Index 2 from Zhou and Xu (2008) for tone differentiability and decomposed it into the differentiable degree of each tone contrast (i.e., tone 1 vs. 2, tone 1 vs. 3, tone 1 vs. 4, tone 2 vs. 3, tone 2 vs. 4, and tone 3 vs. 4). Taking different tones 1 vs. 2 as an example, our algorithm was as follows: assuming *A*_*i*_ represented the intersected area of tonal ellipse 1 and tonal ellipse 2, *A*_1_ and *A*_2_ represented the area of tonal ellipse 1 and tonal ellipse 2, respectively. Then, the differentiability between tone 1 and tone 2 was calculated using the following equation:

$$\text{Differentiability}\left(\text{tone}1\mathrm{vs}.2\right)=\frac{\left(1-\frac{A_{i}}{A_{1}}\right)+\left(1-\frac{A_{i}}{A_{2}}\right)}{2}$$

The differentiability in the 3D method was calculated similarly except that the area of ellipses was changed to the volume of ellipsoids. The center of a tone ellipsoid was placed at the means of the distributions of F0 onset, middle, and offset values of a particular tone and the principal semiaxes were equal to two SDs of the distributions. Tone differentiability became percentage data so that it was more intuitive than the previous index values.

#### Tone Hit Rate

The algorithm of tone hit rate was similar to that of Index 3 in the Zhou and Xu (2008) study. For example, the hit rate of tone 1 was defined as the number of points with tone 1 as the target inside tonal ellipse 1 (or ellipsoid 1) divided by the number of all points (i.e., all tones) inside tonal ellipse 1 (or ellipsoid 1), which was technically the proportion of the points of tone 1 inside tonal ellipse 1 (or ellipsoid 1). In the present study, the proportions of the points of tones 2, 3, and 4 inside tonal ellipse 1(or ellipsoid 1) were also separately calculated to display the hit-rate data in the form of a confusion matrix. The compilation of a hit-rate confusion matrix, which was not conducted in the previous study (Zhou and Xu, 2008), might further provide insight into tone production deficits in prelingually deafened children with CIs.

### Statistical Analyses

The calculated indices in the present study for tone differentiability and hit rate were percentage data and were arcsine transformed before statistical analyses, as the percentage data were not recommended to analyze directly due to the heterogeneous variance. Arcsine transformation was a way to homogenize the variance, making the data more suitable for ANOVA or other statistical analyses (Studebaker, 1985). A two-way ANOVA was conducted to explore the effects of hearing status (i.e., NH or CI) and methods used (i.e., 2D method or 3D method) on the averaged tone differentiability, as well as the averaged tone hit rate. The possible interactions between the main factors were also examined in each two-way ANOVA. A one-way repeated-measures ANOVA was adopted to assess the possible effects of tone types (i.e., tone 1, tone 2, tone 3, or tone 4) on tone hit rate, as well as the effect of tone contrasts (i.e., tone 1 vs. 2, tone 1 vs. 3, tone 1 vs. 4, tone 2 vs. 3, tone 2 vs. 4, and tone 3 vs. 4) on the tone differentiability. In addition, Pearson correlational analyses were conducted for the potential relationship between averaged tone differentiability and hit rate and between the 2D and 3D methods. Pearson correlational analyses were also implemented to examine whether these acoustic indices (tone differentiability and hit rate) were correlated with any of the demographic factors, including age at implantation, chronological age, and CI use duration. In addition, GLM analyses were implemented to examine further the combined contributions of these demographic factors. As chronological age was actually a linear combination of the other two factors (i.e., the sum of age at implantation and duration of CI use), this factor was thus excluded in the GLM. Therefore, the GLM analyses explored the effects of age at implantation, CI use duration, and the interaction of these two main factors on tone production performance.

## Results

### Tone Production Performance in Normal-Hearing and Cochlear Implant Groups

**FIGURE 1:**
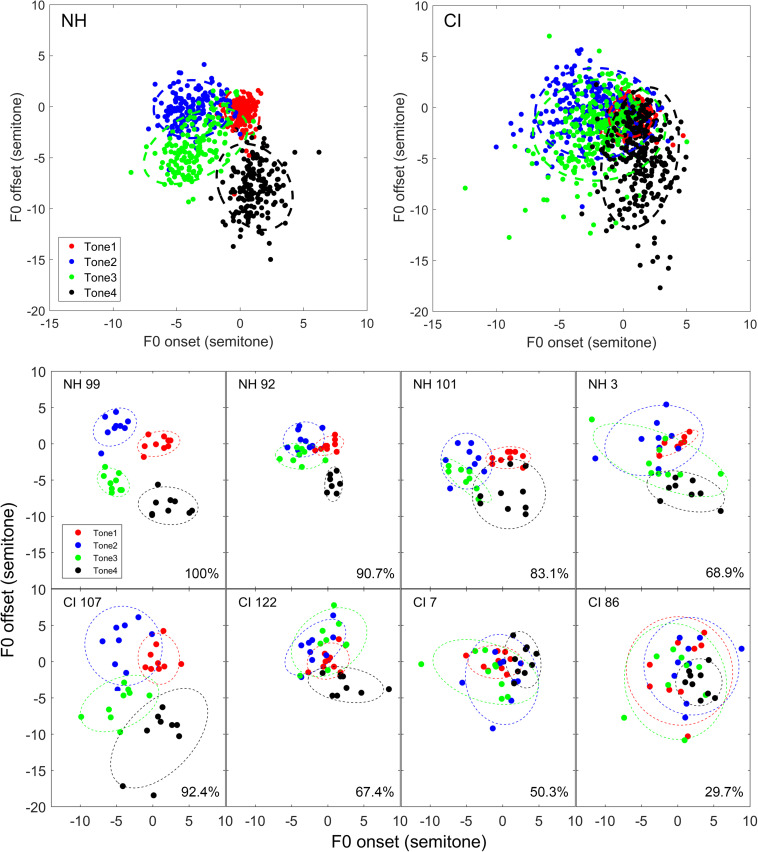
Tone ellipses based on F0 onset and offset values (2D method). Upper panels: Tone ellipses of the NH **(left)** and CI **(right)** groups. Each symbol represents the mean data of one subject. Lower panels: Tone ellipses of individual representative subjects. For either NH or CI group, four subjects were randomly selected based on their mean tone differentiability scores in the interval of 75th–100th, 50th–75th, 25th–50th, and 0–25th quantiles in the respective groups. Mean differentiability score is displayed in the lower right corner. Each data point represents a pair of F0 onset–offset values of a produced token. Four different colors represent four different tone types, as indicated in the legend.

Figure 1 illustrated the tonal ellipses of the two groups based on the 2D method (upper panels) and four representative subjects from either group (lower panels). The representative subjects were randomly selected, one from each quartile of the differentiability score, in respective groups. The boundaries of the four tonal ellipses in the NH group were relatively clearly separated. For tone 1, both F0 onset and offset were relatively high. Thus, the points were mainly located in the upper left quadrant of the scatter plot. For tone 2, the F0 onset was low, and the offset was high, and the data points were mainly located in the upper right quadrant. For tone 3, the heights of both F0 onset and offset were the lowest, making the data points located in the lower-left quadrant. For tone 4, the F0 onset was high, and the offset was low; thus, the corresponding points were located in the lower-right quadrant. Hence, the four ellipses of the NH group were differentiable from each other. However, for the CI group, the scattered F0 data points of the four tones were overlapped with each other to a greater extent. At the individual levels, it was difficult to distinguish some of the tone categories from each other except for the very best performers in the CI group (Figure 1).

**FIGURE 2:**
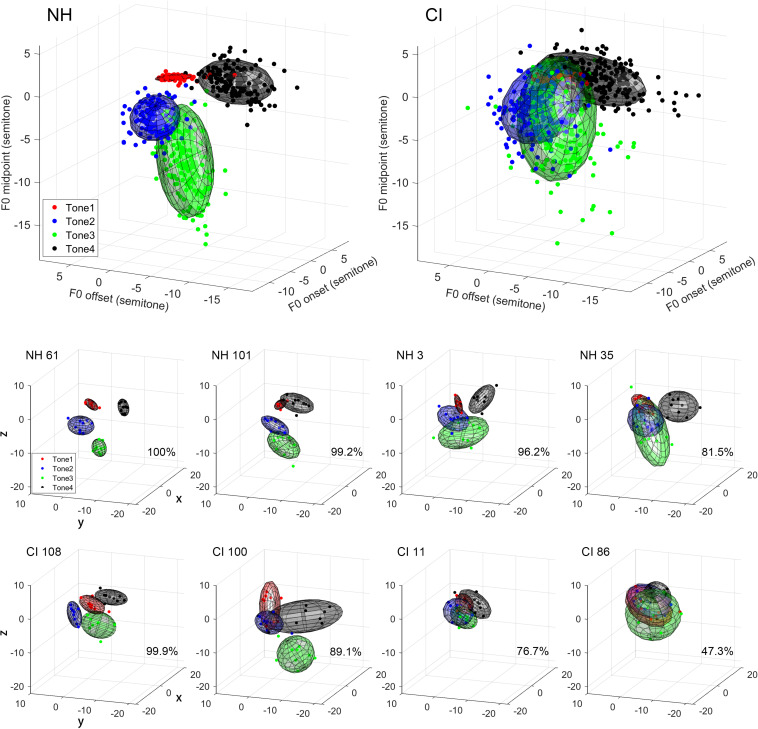
Tone ellipsoids based on F0 onset, midpoint, and offset values (3D method). Upper panels: Tone ellipsoids of the NH **(left)** and CI **(right)** groups. Each symbol represents the mean data of one subject. Lower panels: Tone ellipsoids of individual representative subjects. For either NH or CI group, four subjects were randomly selected based on their mean tone differentiability scores in the interval of 75th–100th, 50th–75th, 25th–50th, and 0–25th quantiles in the respective groups. Mean differentiability score is displayed on the lower right corner. Each data point represents F0 onset–midpoint–offset values of a produced token. Four different colors represent four different tone types as indicated in the legend.

Figure 2 shows the tonal ellipsoids based on the 3D method of the two groups and four representative subjects from either group (lower panels). The representative subjects were randomly selected, one from each quartile of the differentiability score, in respective groups. Like the 2D method, the boundaries of the four tonal ellipsoids in the NH group were relatively clearly separated. The four ellipsoids representing the four tones had their own unique positions in a 3D space and were spatially differentiable from each other. However, the four ellipsoids in the CI group were overlapped with each other to a great degree and were not separable spatially as a whole. At the individual levels, some of the better performers in the CI group showed well-differentiated tonal ellipsoids, and their differentiability scores surpassed those of the poorer performers in the NH group.

### Differentiability of Tone Production in Normal-Hearing and Cochlear Implant Groups

**FIGURE 3:**
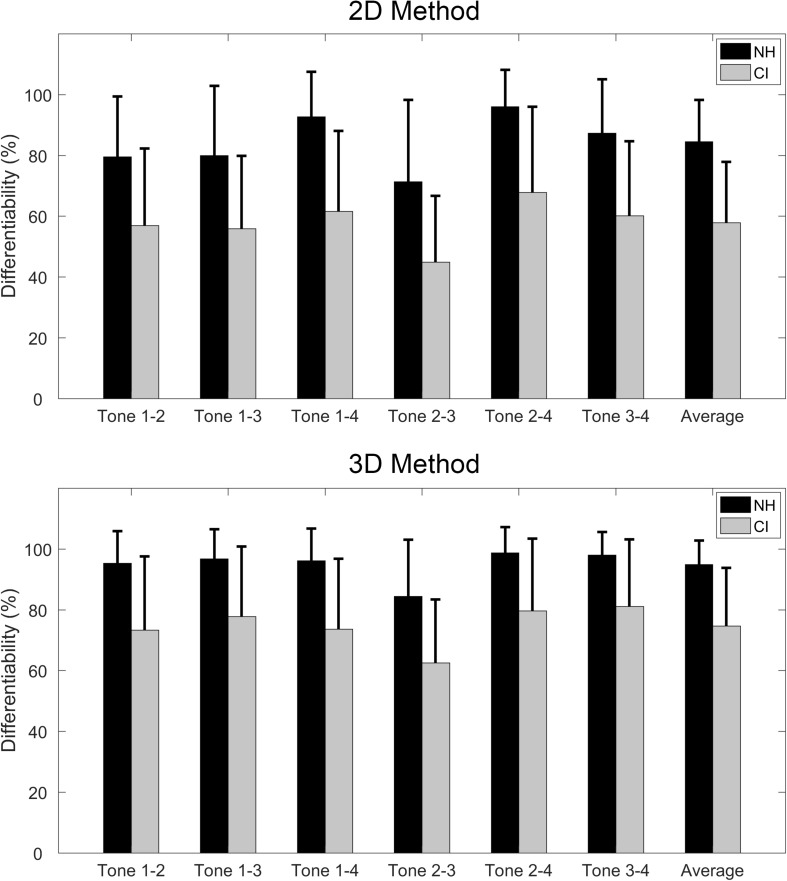
Tone differentiability of the six tone contrasts, as well as the average differentiability, based on the 2D method **(upper panel)** and 3D method **(lower panel)**. Black and gray bars represent the NH and CI groups, respectively. The error bars stand for 1 standard deviation.

The tone differentiability score was computed for each subject to quantify the differentiability of tone contrast in the production. The upper panel of Figure 3 shows the differentiability of each tone contrast based on the 2D method. For the NH group, the differentiability between tone 2 and tone 3 (i.e., contrast tone 2 vs. 3) was the lowest, followed by tone 1 vs. 2 and tone 1 vs. 3. As for the CI group, the differentiability between tone 2 and tone 3 was also the lowest, followed by tone 1 vs. 3 and tone 1 vs. 2. With the 3D method (Figure 3, lower panel), the lowest differentiability was found in tone 2 vs. 3 again for both groups, but there were only minor differences among the other five tone contrasts (i.e., tone 1 vs. 2, tone 1 vs. 3, tone 1 vs. 4, tone 2 vs. 4, and tone 3 vs. 4).

A two-way ANOVA showed significant main effects of both subject group (F = 490.41, *p* < 0.001) and method used (F = 180.68, *p* < 0.001). Additionally, the interaction between these two factors was not significant (F = 0.28, *p* = 0.595). A one-way repeated-measures ANOVA was adopted under each method condition to evaluate further the effects of different tone contrasts on differentiability. For both groups, the one-way repeated-measures ANOVA showed significant differences among the six tone contrasts under both methods (all *p* < 0.001). For the 2D method, *post hoc* comparisons indicated that tone 2 vs. 3 yielded the lowest differentiability score, whereas tone 1 vs. 4 and tone 2 vs. 4 produced higher differentiability scores. For the 3D method, the differentiability score for tone 2 vs. 3 was significantly lower than those of all other tone contrasts.

### Hit Rate of Tone Production in Normal-Hearing and Cochlear Implant Groups

**FIGURE 4:**
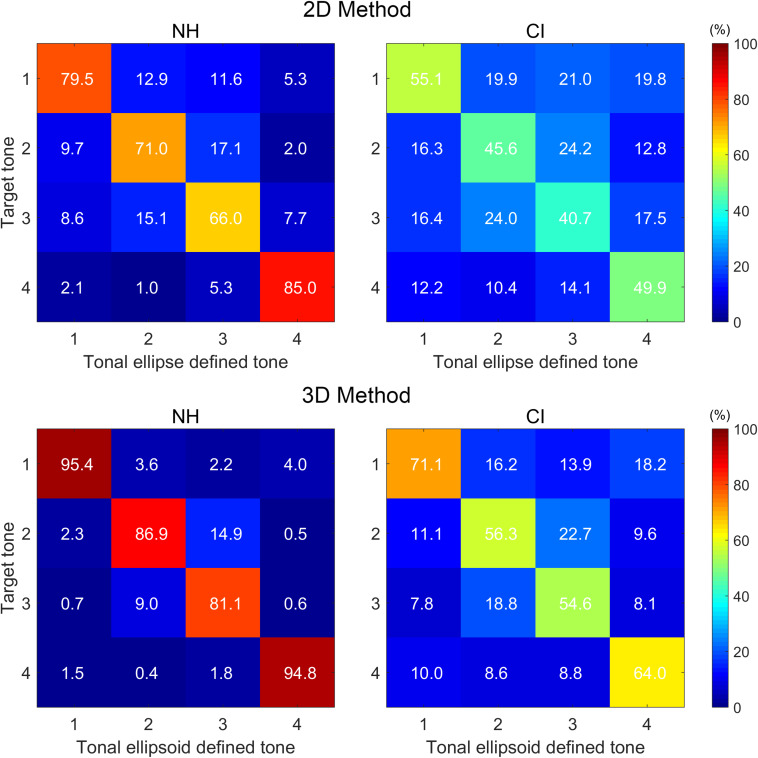
Tone hit-rate confusion matrices of the two groups based on the 2D **(upper panels)** and 3D methods **(lower panels)**. Data were averaged values of all subjects in each group. For each panel of 4 × 4 cells, the columns represent the tone categories defined by the tonal ellipses or the tonal ellipsoids (i.e., within the tonal ellipses or ellipsoids), whereas the rows represent the target tone categories. Value in the cell of row *i* and column *j* is the proportion of target tone *i* inside tonal ellipse (or ellipsoid) *j* (*i* = 1, 2, 3, or 4; *j* = 1, 2, 3, or 4).

Figure 4 shows the confusion matrices of the calculated tone hit rates. A two-way ANOVA revealed significant main effects of both subject group (F = 499.26, *p* < 0.001) and method used (F = 145.08, *p* < 0.001). The interaction between these two factors was not significant (F = 2.98, *p* = 0.084). A one-way repeated-measures ANOVA was performed under each method to evaluate further the effects of different tone types on hit rate. For both groups, the one-way repeated-measures ANOVA revealed significant differences among the four tone types under both methods (all *p* < 0.001). For both NH and CI groups, *post hoc* comparisons indicated that different hit rates among tone types were mainly due to the significantly higher hit rates of tone 1 and tone 4.

### Correlational and Generalized Linear Model Analyses

**FIGURE 5:**
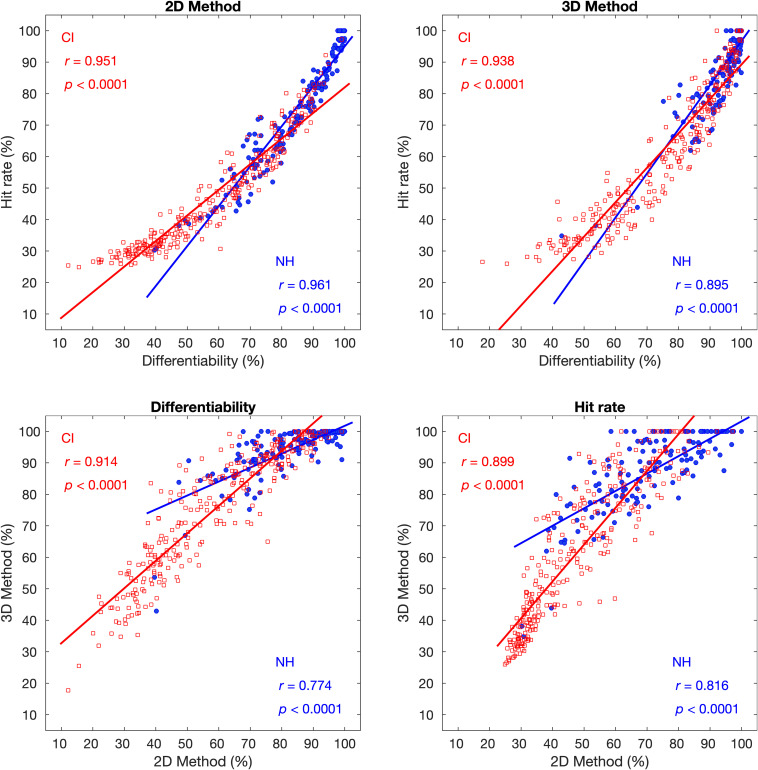
Upper panels: Correlation between averaged tone differentiability and tone hit rate in the 2D **(left)** and 3D methods **(right)**. Lower panels: Correlation between the 2D and 3D methods in tone differentiability **(left)** and hit rate **(right)**. In each panel, each symbol represents one subject. Red open symbols represent subjects in the CI group, and the filled blue symbols represent subjects in the NH group. Red and blue lines are the linear fit of the CI and NH group data, respectively. Correlation coefficient *r* and the corresponding *p*-value for the CI group are shown on the upper left corner, and those for the NH group are shown in the lower right corner.

Pearson correlations were performed between the averaged tone differentiability and hit rate and between 2D and 3D methods. The averaged tone differentiability used here was the average tone differentiability across all six tone contrasts, and the averaged hit rate here was the average value along the diagonal line in the confusion matrix. Figure 5 shows these correlational analyses for the NH and CI groups. The differentiability scores and the hit rates in both NH and CI groups were highly correlated for both 2D and 3D methods (Figure 5, upper panels). In addition, the differentiability scores derived from the 2D and 3D methods were highly correlated with each other (Figure 5, lower left panels). Likewise, the hit rates derived from the 2D and 3D methods were also highly correlated with each other (Figure 5, lower right panels). Note that all these correlations depicted in Figure 5 were statistically highly significant (all *p* < 0.0001).

Pearson correlations were also performed between demographic factors of the CI group (i.e., age at implantation, chronological age, and CI use duration) and acoustic indices (i.e., tone differentiability and hit rate) under both 2D and 3D method conditions. Under both method conditions, age at implantation was significantly negatively correlated with tone differentiability but not correlated with the hit rate. Chronological age was significantly positively correlated with hit rate but not correlated with tone differentiability, whereas CI use duration was significantly positively correlated with both tone differentiability and hit rate. The corresponding correlation coefficients *r* and *p* values are summarized in Table 1. Note that these three demographic factors were not independent of each other. Chronological age was equal to the sum of age at implantation and duration of CI use. Thus, we should interpret these correlations with caution. Although these correlational analyses reveal some significant correlations, the absolute values of the correlation coefficients were small, ranging from 0.165 to 0.343. Note also that the *p*-values in Table 1 are not corrected for multiple comparisons. If we had performed multiple comparisons with Bonferroni correction, then only the correlations related to the duration of CI use would be significant at *p* < 0.05.

**TABLE 1:** The correlation coefficients *r* and *p* values under both Method conditions.

Method	Index	Age at implantation	Chronological age	Duration of CI use
2D	Differentiability	*r* = −0.179 *p* = 0.014*	*r* = 0.105 *p* = 0.152	*r* = 0.291 *p* < 0.001**
	Hit rate	*r* = −0.125 *p* = 0.089	*r* = 0.176 *p* = 0.015*	*r* = 0.339 *p* < 0.001**
3D	Differentiability	*r* = −0.165 *p* = 0.023*	*r* = 0.121 *p* = 0.099	*r* = 0.295 *p* < 0.001**
	Hit rate	*r* = −0.108 *p* = 0.139	*r* = 0.193 *p* = 0.008*	*r* = 0.343 *p* < 0.001**

* indicates a *p* of <0.05, ** indicates a *p* of <0.001.

Age at implantation and duration of CI use was further subject to GLM analyses (Table 2). In these analyses, the duration of CI use but not age at implantation showed significant effects on tone production performance (tone differentiability and hit rate) under both 2D and 3D methods (all *p* < 0.0001). Although the age at implantation was not a significant predictor of tone performance, the interaction of these two factors did play a significant role in the tone production performance of the CI children. The *R*^2^ in the GLM was between 0.154 and 0.189, with both ages at implantation, duration of CI use, and their interactions in the model. These results indicated that age at implantation and CI use duration could jointly explain approximately 15 to 19% of the total variance for the tone production outcomes.

**TABLE 2:** Results of the GLM analyses.

Method	Dependent variable	Independent variable	Coefficient (β)	*t*	*p*	*R*^2^
2D		Age at implantation	0.006	0.631	0.529	0.163
	Differentiability	Duration of CI use	0.050	4.851	<0.0001**	
		A × D^Δ^	−0.007	−2.952	0.004*	
		Age at implantation	0.011	1.266	0.207	0.178
	Hit rate	Duration of CI use	0.046	5.372	<0.0001**	
		A × D^Δ^	−0.006	−3.199	0.002*	
3D		Age at implantation	0.010	0.862	0.390	0.154
	Differentiability	Duration of CI use	0.569	4.773	<0.0001**	
		A × D^Δ^	−0.007	−2.696	0.008*	
		Age at implantation	0.025	1.953	0.052	0.189
	Hit rate	Duration of CI use	0.076	5.833	<0.0001**	
		A × D^Δ^	−0.010	−3.602	0.0004**	

^Δ^A × D stands for the interaction between Age at implantation and Duration of CI use. * indicates a *p* of <0.05, ** indicates a *p* of <0.001.

## Discussion

The present study examined the acoustic properties of tone production in a large group of prelingually deafened children with CIs (*N* = 278). A large group of age-matched children with NH (*N* = 173) was also included as controls. Many acoustic features, such as duration, amplitude contour, and spectral envelope, are associated with lexical tones; however, F0 was the most important acoustic correlate for tones (Xu and Zhou, 2011; Yang et al., 2017b). Based on the F0 data, two methods (2D and 3D) were developed to quantify tone differentiability and tone hit rate of the tone production. Results showed that the 3D method produced consistently higher scores than the 2D method in both tone differentiability and tone hit rate. The children with CIs had much lower scores in tone differentiability and hit rate than the NH children. Tone differentiability in the children with CIs revealed that tone contrast in tone 2 vs. 3 yielded the lowest scores. Tone hit rate revealed that the production of tones 2 and 3 was most often confused with each other in the children with CIs. Both acoustic measures (i.e., average tone differentiability and tone hit rate) showed a weak correlation with duration of CI use, whereas the average tone differentiability showed a weak correlation with the age of implantation. Later, we compare the acoustic findings of the two methods (2D and 3D) and then discuss the tone production proficiency of prelingually deafened children with CIs related to the demographic factors.

There are various ways to evaluate different aspects of Mandarin tone characteristics (e.g., Tupper et al., 2020). Zhou and Xu (2008) used the F0 onset and F0 offset of the F0 contour to analyze the produced tones acoustically. Their methods calculated a series of acoustic indices to assess the overall differentiability among tone types and averaged the hit rate of tones. The present study extended Zhou and Xu (2008) study by expanding the 2D dataset to a 3D dataset, modifying the algorithms of acoustic indices, and greatly enlarging the sample size of the subjects. In Zhou and Xu (2008) study, both Index 1 and Index 2 reflected the overall differentiability among the four tones and significantly correlated with each other (*r* = 0.94, *p* < 0.001). Therefore, there might be some degree of redundancy. In the present study, we modified the algorithm for tone differentiability. We scaled this index into percentage data (see *Methods* for details) to make the index more intuitive. Our modification also allowed us to derive tone contrast differentiability scores. For the tone hit rate, we basically followed the Zhou and Xu (2008) algorithm but further measured the hit rate for each of the four tones rather than just the averaged hit rate. These results were shown in the form of confusion matrices. Another innovation of the present study was that the acoustic indices were calculated based on both 2D and 3D methods, thus making it possible to explore whether the 3D method would better highlight the distinctive characteristics of the tone production of CI children from their NH peers. We expected that the 3D method would improve the scores of the NH group but not the CI group compared with the 2D method, as pediatric CI users tended to produce flat tone contours (Xu et al., 2004; Tang et al., 2019), and the introduction of the middle point of F0 contour would not make any differences on flat tone contours. However, our results showed that the 3D method also improved the scores of the CI group similarly to the NH group (see Figures 3, 4). Further analyses showed that, although the scores of the 3D method were highly correlated with those of the 2D method, the amount of improvement of the 3D method over the 2D method varied greatly (Figure 5). Note that there were extreme cases in which a small proportion of subjects in the CI group either improved as much as >30 percentage points or decreased in scores with the 3D method compared with the 2D method. The two types of extreme cases indicated that errors in tone production might occur at different time segments in the F0 contour. Therefore, the 2D method, combined with the 3D method, might provide useful information to guide the tone rehabilitation process for the hearing-impaired children with CIs.

Through the two-way ANOVA, the two main effects [i.e., (1) NH and CI groups and (2) 2D and 3D methods] on tone differentiability were highly significant. These results indicated that the overall tone differentiability of NH children was significantly better than that of children with CIs and that the 3D method yielded significantly higher tone differentiability scores than the 2D method. It was likely that the 2D method might have underestimated the differentiability among tones, as only two data points (F0 onset and offset) of the F0 contour were used. In addition, we found that no matter what method it was based on, the differentiability of tone 2 vs. tone 3 was always the lowest, both for NH and CI groups. Interestingly, this finding was quite similar to the results of our previous tone-perception study in children with CIs (Mao and Xu, 2017); that is, the recognition rate of the tone 2 vs. tone 3 contrast was the lowest among the six tone contrasts. In addition, the differentiability scores derived from the 3D method were more similar to the absolute values of tone-recognition performance of the six tone contrasts for both NH and CI children. This finding implied that auditory perception was likely to play a decisive role in the acquisition of tone production and that the 3D method might be more precise in reflecting the true tone differentiability.

For tone hit rate, it was observed that the two main effects [i.e., (1) NH and CI groups and (2) 2D and 3D methods] on tone hit rate were also significant. These results illustrated that the averaged hit rate of the NH group was significantly higher than that of the CI group, and the averaged hit rate calculated based on the 3D method was significantly higher than that based on the 2D method. Under either method, the error pattern of tone production was similar to the tone-perception error pattern, except that the values of the diagonal in the tone-production matrices (Figure 4) were, in general, lower than that of the tone-perception matrices (Mao and Xu, 2017). Confusion of tone 2 and tone 3 with each other was the most prominent error for both NH and CI children. This was consistent with the findings for tone differentiability. Several earlier studies had found that the F0 contours of the four tones produced by pediatric CI users tended to be flat (Xu et al., 2004; Tang et al., 2019). This may be largely related to pitch perception deficits in CI users. Therefore, when CI children do not perceive the F0 contours of all tone types, their production tends to be flat with little pitch variation across the duration of the syllables. The flat pitch production was exacerbated when prelingually deafened children with CIs were asked to sing a song (Nakata et al., 2006; Xu et al., 2009; Mao et al., 2013), although some of them can achieve normal pitch production after rigorous, long-term training (Yang et al., 2019).

The present study analyzed the potential relationships within the acoustic indices. Not surprisingly, the average tone differentiability scores and the average hit rate were highly correlated for both groups (Figure 5, upper panels), similar to the findings among different indices in Zhou and Xu (2008) study. Although these two metrics were highly correlated, they provided insights into different aspects of tone production. Tone differentiability focused on the tone contrast, whereas the hit rate allowed us to construct the tone confusion matrix. For both acoustic measures, the scores between 2D and 3D methods were also highly correlated in both groups (Figure 5, lower panels). In the CI group, the 3D method yielded scores, on average, 16.6 and 13.6 percentage points higher than those of the 2D method for tone discrimination and hit rate, respectively. The higher scores produced by the 3D method might correspond more closely to the tone-perception outcomes of the CI children (Mao and Xu, 2017). However, the strong correlation between the scores of the 2D and 3D methods suggests that the simpler, 2D method might be efficient in clinical practice, whereas the 3D method might provide complementary information for an acoustic assessment of lexical tones.

There is abundant evidence showing that tonal ability of pediatric CI users is correlated with age at implantation or device use duration (Peng et al., 2004; Han et al., 2007, 2009; Lee et al., 2010; Xu et al., 2011; Zhou et al., 2013; Li et al., 2017; Mao and Xu, 2017; Tang et al., 2019), although the literature is not always consistent on the contributions of these two predictors. For example, Peng et al. (2004) and Tang et al. (2019) found that age at implantation was the only significant predictor for tone production, whereas Han et al. (2007) revealed that CI use duration also significantly predicted the tone production ability. For tone perception, several studies showed that age at implantation exerted a weak effect on CI users’ tone perception ability, whereas the duration of CI use seemed to be a more robust predictor (Zhou et al., 2013; Li et al., 2017). An earlier study by Wong and Wong (2004) did not show any correlations of Cantonese tone identification with either implantation age or duration of CI use. However, more recent evidence encouraged early implantation for children with severe to profound sensorineural deafness for tonal ability rehabilitation (Peng et al., 2004; Han et al., 2007, 2009; Lee et al., 2010; Xu et al., 2011; Zhou et al., 2013; Li et al., 2017; Mao and Xu, 2017; Tang et al., 2019). Our results from the present study with a fairly large sample of subjects showed that duration of CI use was a significant predictor for both tone differentiability and hit rate, whereas age at implantation and chronological age seemed to be the weaker predictors, as they were only correlated with one of the indices (Table 1). Some of our CI participants who were implanted at an earlier age happened to be at a younger chronological age at the test, which could counteract part of the benefit of earlier implantation. The GLM analyses demonstrated that in the presence of duration of CI use, the effect of age at implantation was not manifested, but it had a significant interaction with a duration of CI use, and jointly, these two factors accounted for approximately 15–19% of the total variance of tone production performance of the CI children. Interestingly, for tone perception ability, earlier evidence had shown that these two variables could jointly explain approximately 50% of the outcome variance (Xu and Zhou, 2011). The difference of more than 30% of the variance interpretability between perception and production by the temporal factors might reside in the differences in the time course of development of perception and production, among other non-temporal factors. Generally, our results illustrated that persistent CI use might play a key role in developing vocal production of Mandarin tones for those implanted children. Besides lexical tone-related aspects, previous studies also supported the persistent use of CI devices for the music-related development of pediatric implantees (Yucel et al., 2009; Chen et al., 2010; Mao et al., 2013). The effects of duration of use could be attributed to their maturity, persistent training and learning, and increased experiences with time. Overall, our results supported early implantation and continual use of CI devices in the tone rehabilitation process of pediatric CI users. It is noteworthy that, compared with the NH peers, the children with CIs might still demonstrate deficits in tone production even after prolonged use of the devices despite the significant progress in CI technology in recent years.

## Conclusion

The present study modified a previous 2D method and developed a new 3D method to assess lexical tone production in children with CIs. Two acoustic measures (i.e., tone differentiability and hit rate) were derived from the 2D and 3D methods. With a relatively large sample size, our results confirmed that the tone production ability of the CI children was significantly inferior to that of the normally developed children in both tone differentiability and hit rate. The scores obtained with the 2D and 3D methods were highly correlated, suggesting that the simpler, 2D method would be efficient in capturing the main acoustic characteristics of tone production and might be more practical for clinical assessment of lexical tone production. However, the 3D method might provide complementary information for the tone production deficits when combined with the 2D method. The tone differentiability and hit rate, although capturing different aspects of tone production, were also highly correlated with each other. Age at implantation and especially the duration of CI use were important predictors for tone-production ability but could only account for 15 to 19% of the variance. Other factors such as rehabilitation or training on tone production, mother’s education, children’s IQ, residual hearing, etc., should be explored in future studies of tone development in prelingually deafened children with CIs.

## Data Availability Statement

The raw data supporting the conclusions of this article will be made available by the authors, without undue reservation.

## Ethics Statement

The studies involving human participants were reviewed and approved by The Institutional Review Board of Ohio University. Written informed consent to participate in this study was provided by the participants’ legal guardian/next of kin.

## Author Contributions

YM and LX conceived the study, designed the analysis, and wrote the manuscript. LX collected the data. YM, SX, and HC obtained funding for this research. All authors discussed the results and implications and commented on the article at all stages.

## Conflict of Interest

The authors declare that the research was conducted in the absence of any commercial or financial relationships that could be construed as a potential conflict of interest.
